# Fully automated F-wave corridor extraction and analysis algorithm for F-wave analyses and MUNE studies

**DOI:** 10.1038/s41598-023-41183-5

**Published:** 2023-08-24

**Authors:** N. Tuğrul Artuğ

**Affiliations:** Department of Electric, Vocational School of Technical Sciences, Istanbul University-Cerrahpasa, Buyukcekmece, Istanbul, Turkey

**Keywords:** Neurology, Biomedical engineering, Electrical and electronic engineering, Motor neuron disease

## Abstract

F-waves are used in motor unit number estimation (MUNE) studies, which require rapid dedicated software to perform calculations. The aim of this study is to define a mathematical method for a fully automated F-wave extraction algorithm to perform F-wave and MUNE studies while performing baseline corrections without distorting traces. Ten recordings from each class, such as healthy controls, polio patients and ALS patients, were included. Submaximal stimuli were applied to the median and ulnar nerves to record 300 traces from the abductor pollicis brevis and abductor digiti minimi muscles. The autocorrelation function and the signal of sum of all traces were used to find the location for the maximum amplitude of the F-waves. F-waves were revealed by using a cutting window. Linear line estimation was preferred for baseline corrections because it did not cause any distortion in the traces. The algorithm automatically revealed F-waves from all 30 recordings in accordance with the locations marked by a neurophysiologist. The execution of the algorithm was less than 2 (usually < 1) minutes when 300 traces were analyzed. Mean sMUP amplitudes and MUNE values are important for differentiating healthy controls from patients. Moreover, F-wave parameters belonging to polio patients on whom there was a relatively low number of studies conducted were also evaluated.

## Introduction

F-waves are late responses that can be recorded by the stimulation of peripheral nerves. It is recorded after compound muscle action potential (CMAP), which is known as the M-response. To obtain the M-response, peripheral nerves are stimulated, and the stimulation level is increased until the peak value in the amplitude is reached. This type of stimulation is called supramaximal stimulation.

The M-response includes the sum of all action potentials^1^. While the triggered action potentials invade the lower motor neurons’ cell body toward dendrites, some of them can backfire, and regenerated action potentials reach the examined muscle. It is possible to record this activity as an F-wave over the target muscle. They appear on the EMG instrument monitor later than the M-response^1,2^.

F-waves consist of a reduced combination of action potentials that are all included in the M-response. The latency of F-waves differs according to the length of the pathway that is determined by the muscle selected for recording. Regarding muscles innervated by median and ulnar nerves, F-waves are not elicited before 20 ms^3,4^.

The shape and latency of F-waves change across stimuli because the contribution of action potentials to the recorded waveform differs. However, it is possible to observe F-waves with the same morphology in consecutive traces if at least 90 traces are recorded^2^. If a pair of F-waves has the same amplitude, shape and latency, they are called repeater F-waves^2^.

Two types of stimulations are used to elicit F-waves. One stimulation is the supramaximal level, and the other is the submaximal level^2^. Using supramaximal stimulus produces F-waves with maximum persistence. On the other hand, the purpose of submaximal stimulation is to decrease the number of F-wave combinations. Thus, the probability of observing repeater F-waves increases. To perform an F-wave study by using the submaximal stimulus, first of all, maximum amplitude for the M-response should be adjusted by applying supramaximal stimulation. Then, the stimulation level should be decreased to 30–50% of the maximum amplitude of the M-response.

While recording consecutive traces, sometimes an F-wave is not observed on a trace. It is possible to encounter this situation in healthy controls, but it is especially observed in disease models with motor unit denervation^5^. The action potential pool that forms the M-response decreases after motor unit denervation, so the number of different F-waves decreases. As a result, the possibility of observing repeater F-waves increases^6^.

In F-wave studies, some parameters are extracted for analysis. These are the number of repeater F-waves (total number of repeater F-waves), number of repeater neurons (number of different repeater F-waves), sMUP amplitude (peak-to-peak amplitude of repeater F-wave), M-response amplitude (peak-to-peak amplitude of M-response), persistence (ratio of number of noticeable F-waves to total number of recorded traces), latency (the latency between M-response and F-wave) and duration of F-waves^1,2^.

Standard parameters related to F-waves are calculated by neurologists, and this process is too time-consuming. There is a necessity to develop an algorithm to perform these calculations and to reduce the load on examiners. In addition, dealing with many traces (90–300) to perform F-wave analysis for a neurologist is very difficult. It is possible to miss some repeater F-waves while performing manual inspection. However, a computer-aided algorithm will increase the accuracy of the analysis.

While observing the progression of motor neuron diseases, it is important to estimate the remaining number of motor units of a patient. Many motor unit number estimation (MUNE) methods have been introduced in the literature. Incremental stimulation^7^, multiple point stimulation^8^, spike-triggered averaging^9^, adapted multiple point stimulation^10^, F-MUNE^11^, statistical MUNE^12^, high-density surface EMG^13^, MUNIX^14^ and MScanFit^15^ are the prominent techniques. The calculation of the M-response parameters is the same for all methods, but it is the sMUP calculation that makes a difference among the MUNE calculation methods.

Some studies that introduce algorithms for calculating F-wave parameters along with MUNE calculations have been conducted^11,16–19^. It is clear that F-waves can be used to calculate MUNEs, but a dedicated algorithm should be used for this purpose^20^.

While studying F-waves, it is challenging to determine the baseline of the F-wave. There are several kinds of baselines on which the signal rides, such as linear, exponential, polynomial or sigmoidal baselines^21^. Sometimes, F-waves elicit close to M-responses^11^, and their baseline should be corrected to have a better accuracy. Widely accepted baseline correction algorithms are polynomial fitting (PF)^22^, small window moving average (SWMA)^23^ and adaptive iteratively reweighted penalized least squares (AIRPLS)^24^.

An action potential recording on a trace harbors the sum of several MUAPs. Baseline corrections without changing the waveform of recordings for electromyography signals are a crucial matter because valuable information under the peaks of action potentials might be lost.

The present study will explain how to properly extract the F-wave corridor from consecutive traces for F-wave and MUNE studies. F-wave corridor extraction is an isolation procedure to reveal F-waves while discarding the other parts of the traces including M-response. It simplifies the analysis process from the viewpoint of software because the number of samples that represents a trace is reduced up to 30% of the original one.

The developed algorithm, which has baseline correction features, will be applied to recordings of healthy controls, ALS patients and polio patients, for whom a few studies dealing with F-waves are present. The new algorithm retains valuable information under the peaks without distorting the traces, and it performs the MUNE calculation faster than the other algorithms by using F-waves.

The aim of this study is to:

- Define a fully automated mathematical method for the proper extraction of F-waves while performing baseline corrections without distorting the recorded traces.
- Perform faster MUNE calculations with recorded F-waves.
- Determine the differentiating F-wave features to distinguish among healthy subjects, ALS patients and polio patients.

This paper continues with material and methods. Information regarding the subjects in the dataset and details regarding the data acquisition procedure are provided in this section. The Theory/Calculation section demonstrates the newly developed algorithm with detailed graphics. The results, which are supported by statistical tests, are shared in the next section. In the discussion section, prominent findings are provided, and the current study is compared with other studies. The paper ends with a conclusion.

## Materials and methods

The study was approved by the local ethics committee of Istanbul University, Istanbul Faculty of Medicine (2016/162). All experiments were performed in accordance with relevant guidelines and regulations. Informed consent was signed by all participants of this study.

### Subjects

In the dataset, there are 10 recordings of each class. They are amyotrophic lateral sclerosis (ALS) patients, polio patients and healthy controls.

The mean age of the ALS patients (5 males/5 females) was 56.5 (32–80). The ALS patients were classified according to the Awaji criteria as definite in 8, possible in 1, and probable in 1. The mean MRC sum score and ALSFRS-R score were 52 ± 5.8 and 39 ± 5.8, respectively.

The polio patients were 5 males and 5 females. Their mean age was 55.3 (37–76). The mean MRC sum score of the patients with poliomyelitis patients was 49.3 ± 6. Regarding the healthy controls (3 males/7 females), the mean age was 39.5 (31–62).

### Electrophysiological evaluation

The recording procedure was briefly explained to participants. Their recording muscles were in a resting state, and their arms were backed up with a pillow. They were lying down on their back for the recording process. It was proposed to participants that they remain steady to reduce movement artifacts.

The skin temperature of the recording muscle was maintained at 33 °C or over. Skin temperature was measured by using an infrared thermometer before recording. If it was below 33 °C, hot packs were used to increase the skin temperature over 33 °C. Periodical temperature measurements were done during recording process to ensure that the skin temperature was over 33 °C. If it was needed, hot packs were used again to increase the skin temperature.

A Medelec Synergy EMG instrument (Natus Medical, USA) was used to acquire signals. The cut-off frequency for the bandpass filter was 20 Hz–10 kHz. The amplifier gain was adjusted to 500 μV for an optimal view of the traces. The time axis was adjusted to 10 ms/division. The sampling frequency of the EMG instrument was 50 kHz, and the trace duration was 100 ms.

Disposable pre-gelled and 20 mm sized Ag/AgCl surface electrodes were used for recording CMAPs and F-waves as well as for stimulation. CMAPs were recorded by using submaximal stimuli (30–50% of the maximum CMAP amplitude) with 0.5 Hz frequency^11^.

**Figure 1:**
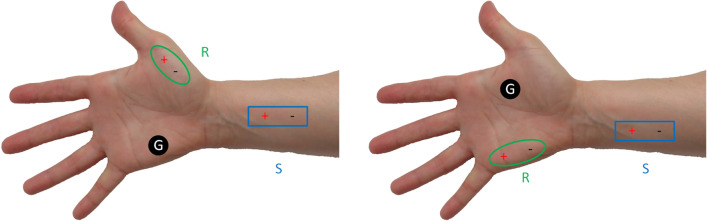
Electrode placements for median (left) and ulnar (right) nerve recording. *R* recording electrode, *S* stimulator, *G* ground.

The median nerve was stimulated at the wrist to perform recording from the abductor pollicis brevis (APB) muscle. The abductor digiti minimi (ADM) muscle was selected to perform the same procedure for the ulnar nerve. The cathode was placed proximal to the anode for the stimulator. Recording electrode was placed over muscle according to belly-tendon recording principle^25^. Anode of the recording electrode was placed near the tendon of the recording muscle. Cathode of the recording electrode was placed over muscle belly, corresponding to the neuromuscular junction. The ground electrode was placed to the palm for both recording setups. Figure 1 shows the electrode placements for recording procedure.

In each session, 300 consecutive traces were recorded for each muscle. Two criteria were applied in the study. First, the amplitude of the CMAP had to be greater than at least 1 mV. Also, only F-waves with amplitudes greater than 40 µV were accepted for analysis. By following the recording criteria, one recording per muscle was performed for one person. It took 10 min to prepare a person, to configure the EMG instrument and to place electrodes. Recording from one muscle took 10 min. The total session duration of one person was nearly 30 min.

### Statistical analysis

Many statistical analysis methods to be performed on data are based on the assumption that the data are normally distributed. Studying on data that do not have a normal distribution affects the validity of statistical tests and the reliability of analysis results. While parametric tests give more reliable results, non-parametric tests are preferred if the data are not normally distributed.

Normal distribution tests were performed for all calculated features. Histogram, skewness and kurtosis values, detrended normal Q–Q graphics, and Shapiro‒Wilk normality tests were evaluated for this purpose.

Histogram is a graphic that shows the distribution of data with bars. For normal distribution, a bell curved graphic is expected.

Skewness and kurtosis values should be close to 0 for a normal distribution. If kurtosis value is positive, that means the distribution has fatter tails with a sharper peak. If kurtosis value is negative, that means the distribution has thinner tails with a flatter peak. If skewness value goes out of ± 1, that means data have a right or left skewed distribution according to the sign of this value.

Detrended normal Q–Q graphic is a type of Quantile–Quantile plot which demonstrates the distribution of data in a different way. Samples of the data should be scattered on the graphic for having a normal distribution. If there is some clustering in this plot, it means that the data may be right or left skewed.

Shapiro‒Wilk test is used for data which have number of samples less than 30. Significance value should be greater than 0.05 to have a normally distributed data.

If a feature was not normally distributed as a consequence of these tests, logarithmic transform was applied to this feature, and all these tests were repeated. As a result, since all features were normally distributed, all groups were compared for each feature by using parametric ANOVA. Afterward, post hoc analysis was performed with Tukey’s test.

## Theory/calculation

In this section, the automated algorithm is introduced by using healthy control recordings. The recorded signals acquired by using the EMG instrument are extracted to a text file. This file contains participant information, EMG instrument settings, etc., with the numerical data. It should be purified from redundant data by using pre-processing techniques, and the necessary part from the signal recording is saved to an Excel sheet.

**Figure 2:**
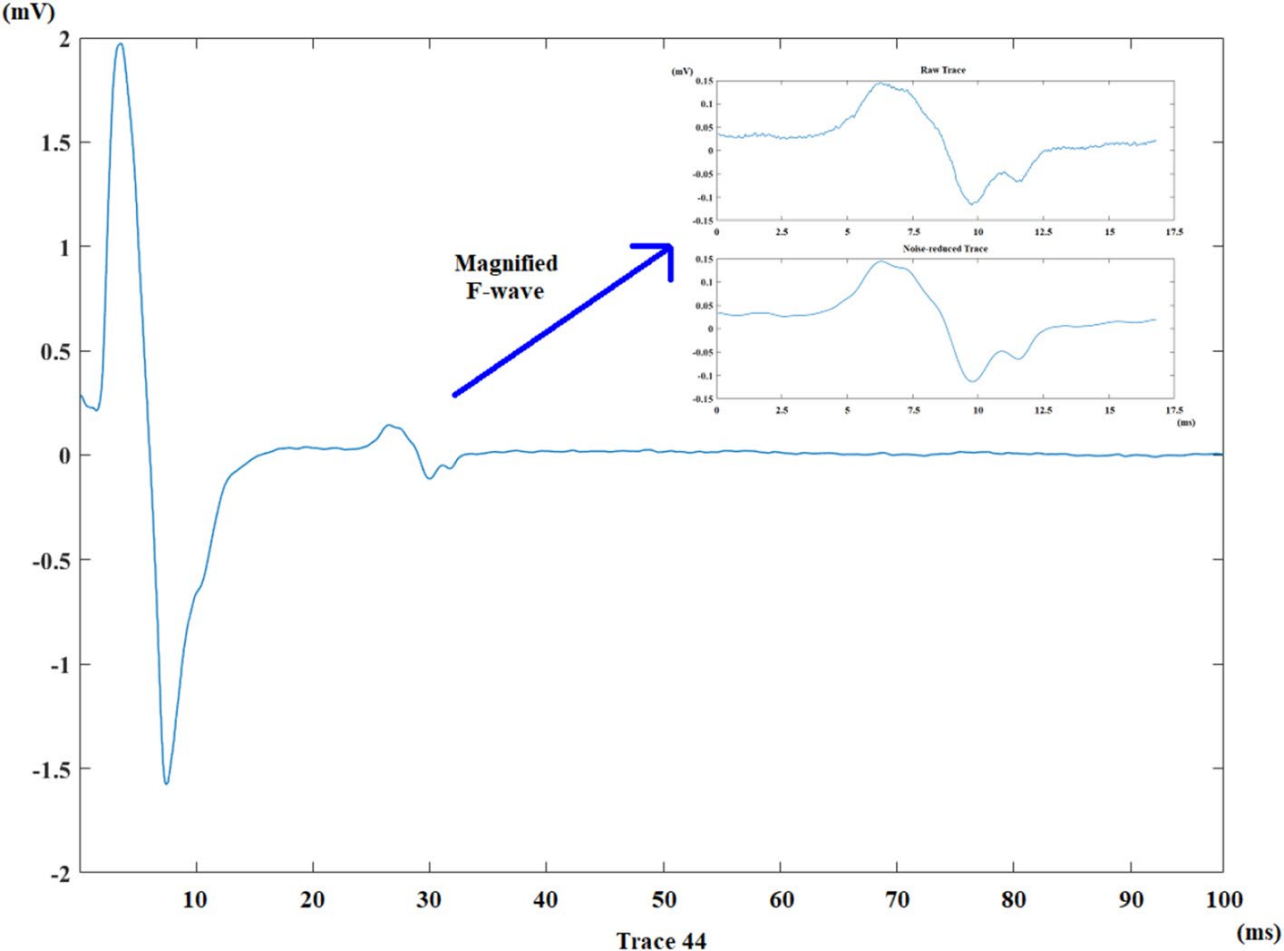
An entire trace from the median nerve recording of a healthy control (stimulus artifact was discarded) and the result of noise reduction detail of the F-wave belonging to the same trace.

The Excel file belonging to a signal recording is read by the currently developed algorithm. The noise on all traces is reduced by a wavelet-based algorithm. It has been seen that the Daubechies 12 wavelet provides the best result for this kind of signal from previous studies^26^. Figure 2 demonstrates an entire trace from a healthy control recording and the result of noise reduction on the F-wave for this trace.

**Figure 3:**
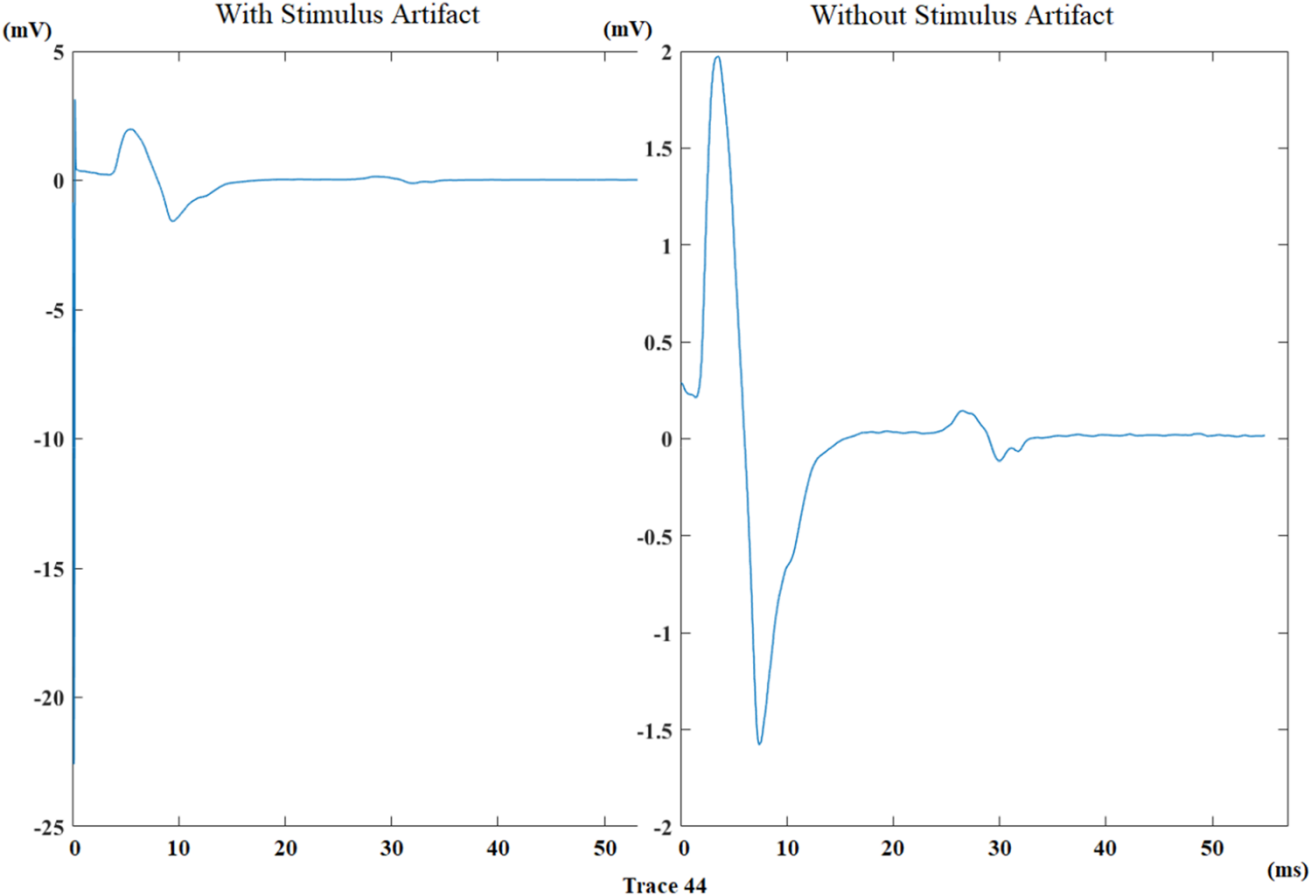
A trace from the median nerve recording of a healthy control before and after discarding stimulus artifact.

The duration of the recorded traces is 100 ms. At the very beginning of these traces, there may be stimulus artifacts, so 2 ms from the beginning of each trace is discarded. A trace is shown in Fig. 3 before and after discarding stimulus artifact. Stimulus artifacts shrink the signals in terms of appearance on the monitor, and after discarding them, the M-response and F-wave can be observed clearly on the right-hand side of Fig. 3.

After this operation, an option is offered to the user to continue either a fully automated analysis or a manual analysis. If the manual option is selected, the user can individually check all traces and discard any of them before starting the analysis. The user can determine the F-wave corridor manually by entering left and right cutting locations. Then, the algorithm continues to the baseline correction process. If the fully automated option is selected, the algorithm begins directly to find the F-wave corridor.

**Figure 4:**
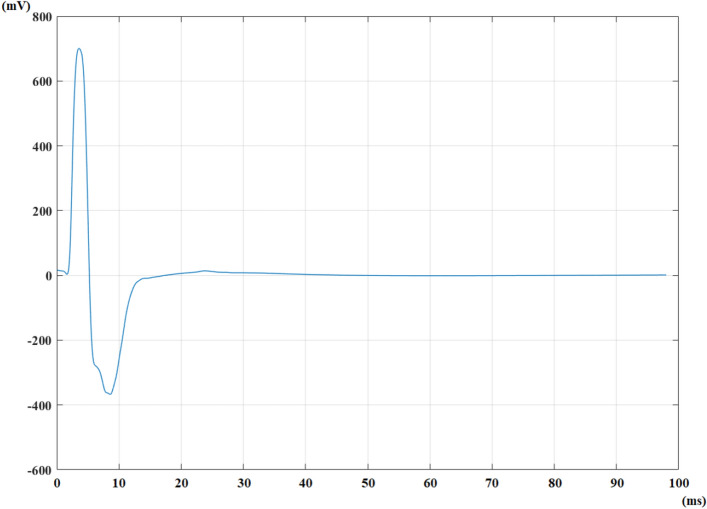
The signal of sum of all traces for a healthy control from a median nerve recording.

In the case of selecting the fully automated option, all traces are summed, and the autocorrelation function is calculated. The signal of sum of all traces for a healthy control can be seen in Fig. 4.

Calculation of autocorrelation function for an EMG signal is similar to convolution operation with one difference. In this calculation, second signal is not flipped while shifting it from one side to another. Autocorrelation measures the similarity between a vector and the shifted copy of the same vector.

The autocorrelation function is helpful for determining the cutting location of the area of interest from the signals^27^. This function is calculated by using the signal of sum of all traces. Because all traces are summed, M-response has the highest amplitude peak and this peak is always the first peak. The second highest amplitude peak is formed because of the F-waves which are elicited on similar locations. It is possible to observe other peaks because of the nature of F-waves. but their amplitudes are lower when they are compared with the first and the second highest amplitude peaks. This valuable information is used to find the exact maximum amplitude location of F-waves on the signal of sum of all traces.

The location of the global maximum is searched at the 7.5 ms part of the signal of sum of all traces.

The second peak from the autocorrelation function is marked. If the distance between the first peak and the second peak is less than 12 ms, it means that there is some fluctuation in the M-response, and there is an artificial peak in the autocorrelation function. Therefore, the next peak on the autocorrelation function is selected.

**Figure 5:**
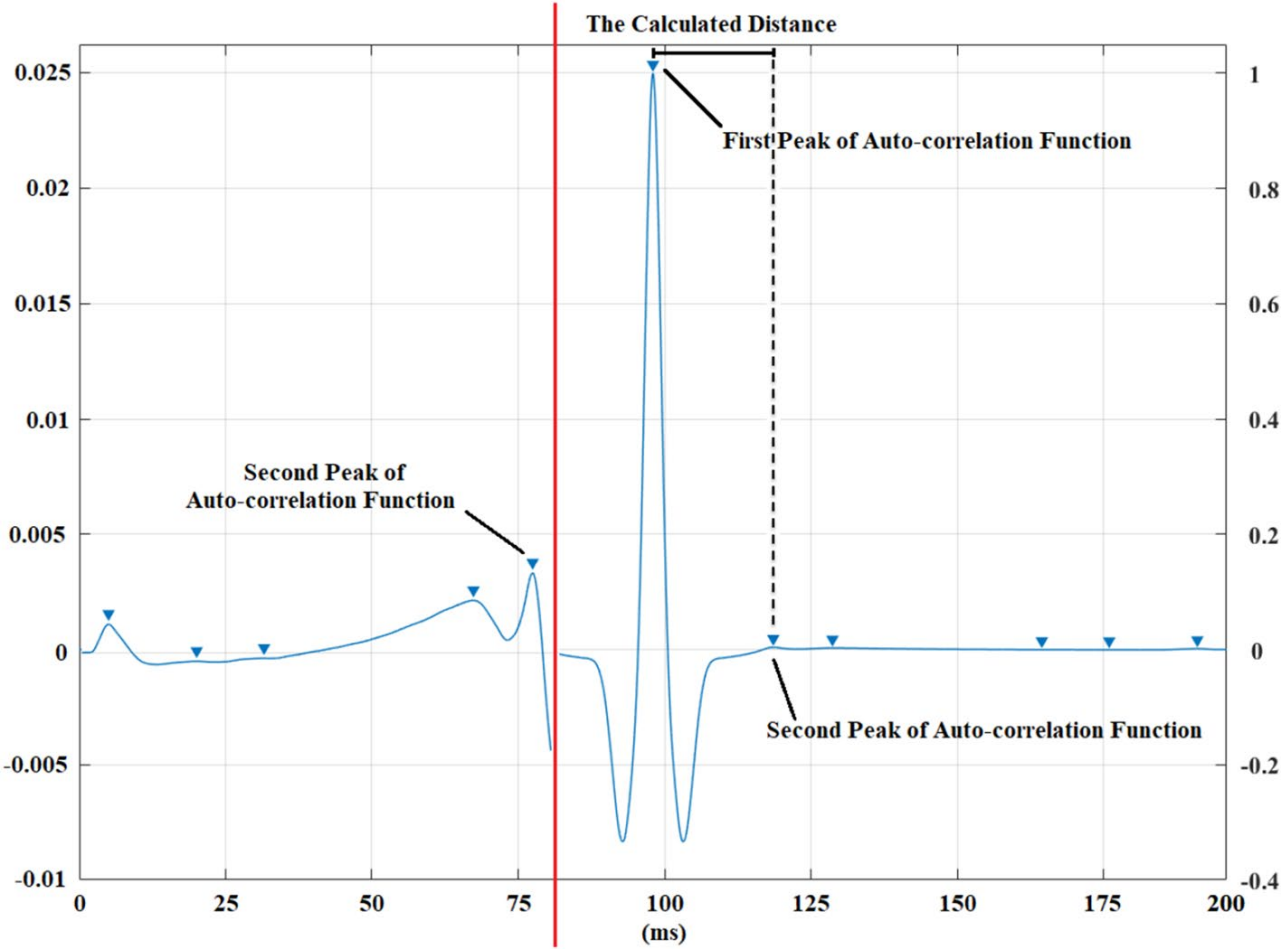
The first and the second peak of the autocorrelation function and the calculated distance between the locations of the global maximum and the second peak at the autocorrelation function for a healthy control from a median nerve recording [triangles: peaks] (left side of the red line was constructed by breaking the ordinate to observe the peaks clearly).

The distance between the locations of the first peak and the second peak at the autocorrelation function is calculated. After adding this calculated distance to the location of global maximum at the signal of sum of all traces, the location of the maximum amplitude for the F-wave is searched ahead of this distance. The first and second peak from the autocorrelation function and the calculated distance belonging to a healthy control recording can be seen in Fig. 5.

Left part of Fig. 5 was constructed by breaking the ordinate to observe the second peak and the other peaks clearly. It must be noted that the calculated distance is not the exact location; it is an approximate location for calculating F-wave corridor cutting locations.

**Figure 6:**
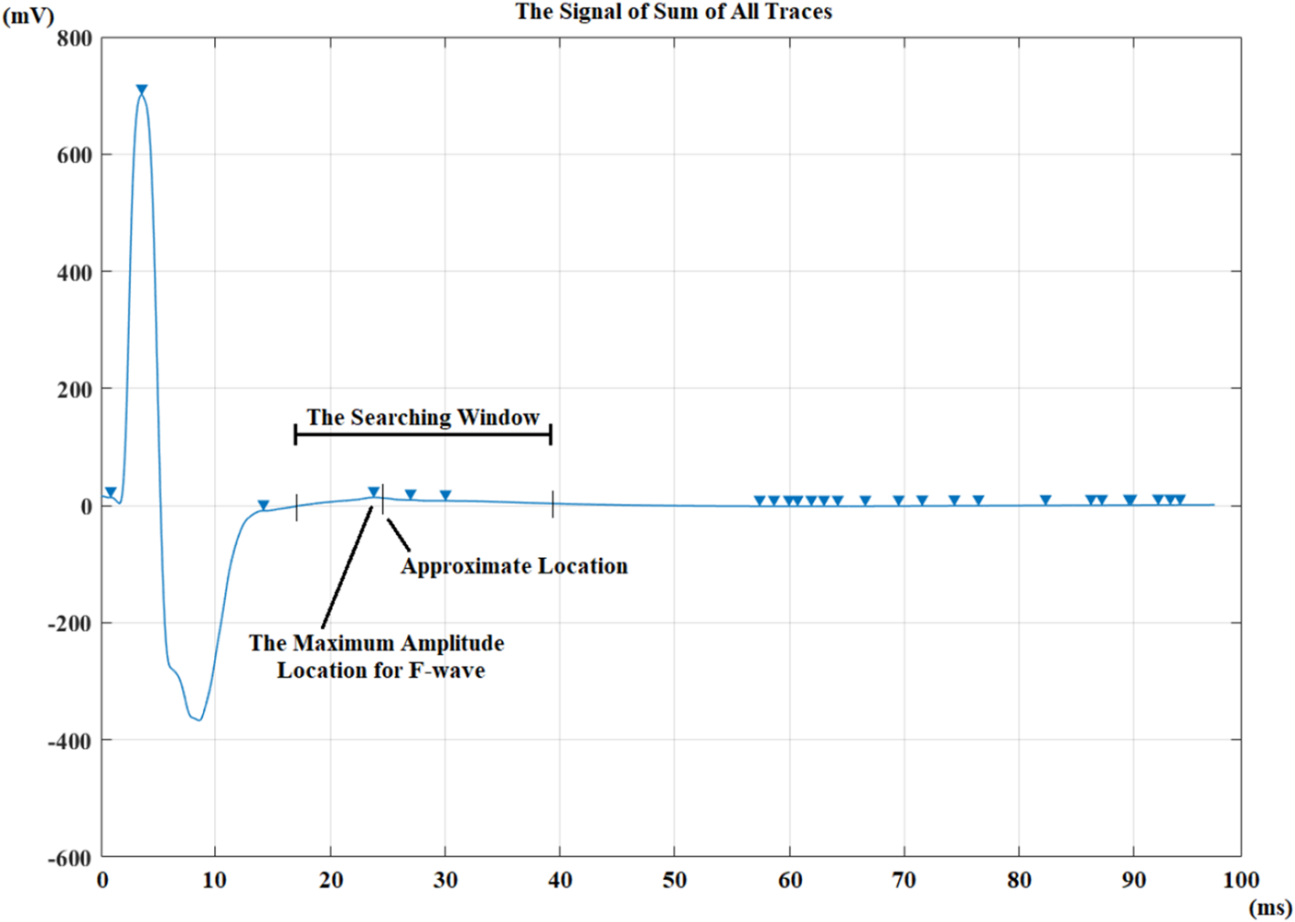
The maximum amplitude location searching window, approximate location for maximum amplitude location of F-waves which is acquired from autocorrelation function and the exact maximum amplitude location for the F-waves belonging to a healthy control recording [triangles: peaks].

A signal is extracted using a window by moving back 7.5 ms and forward 15 ms from the approximate location at the signal of sum of all traces (Fig. 6). The positive peak inside the 22.5 ms searching window that has the maximum amplitude is the location for the maximum amplitude location of the F-waves. If there is no positive peak in this window, the next peak is selected from the autocorrelation function, and all the previous steps are repeated. The searching window, the approximate maximum amplitude location of F-waves and the exact maximum amplitude location for the F-waves are depicted in Fig. 6.

If the maximum amplitude location for the F-wave is calculated near the global minimum because of the fluctuation in the M-response, the amplitude at this location is below at least − 100 mV at the signal of sum of all traces. In a situation such as this, the next peak on the autocorrelation function is selected, and all previous steps are repeated.

F-waves are not elicited before 20 ms on the median or ulnar nerves^3,4^. If the maximum amplitude location for the F-wave is earlier than 16 ms, the next peak on the autocorrelation function is selected, and all previous steps are repeated. This threshold was determined as 16 ms, but it is actually 18 ms in raw signal because 2 ms part was discarded as stimulus artifact.

A sample signal is extracted by moving 7.5 ms back and by moving 7.5 ms forward in the signal of sum of all traces from the maximum amplitude location for the F-waves. If the slope of this 15 ms sample signal is greater than 16°, then the left part of this signal comes from the M-response. This value of 16° has been determined as the ideal value of the result of many trials with numerous recordings. After the last examination, the next peak on the autocorrelation function is selected if the slope of the signal is greater than 16°, and all previous steps are repeated for the last time.

**Figure 7:**
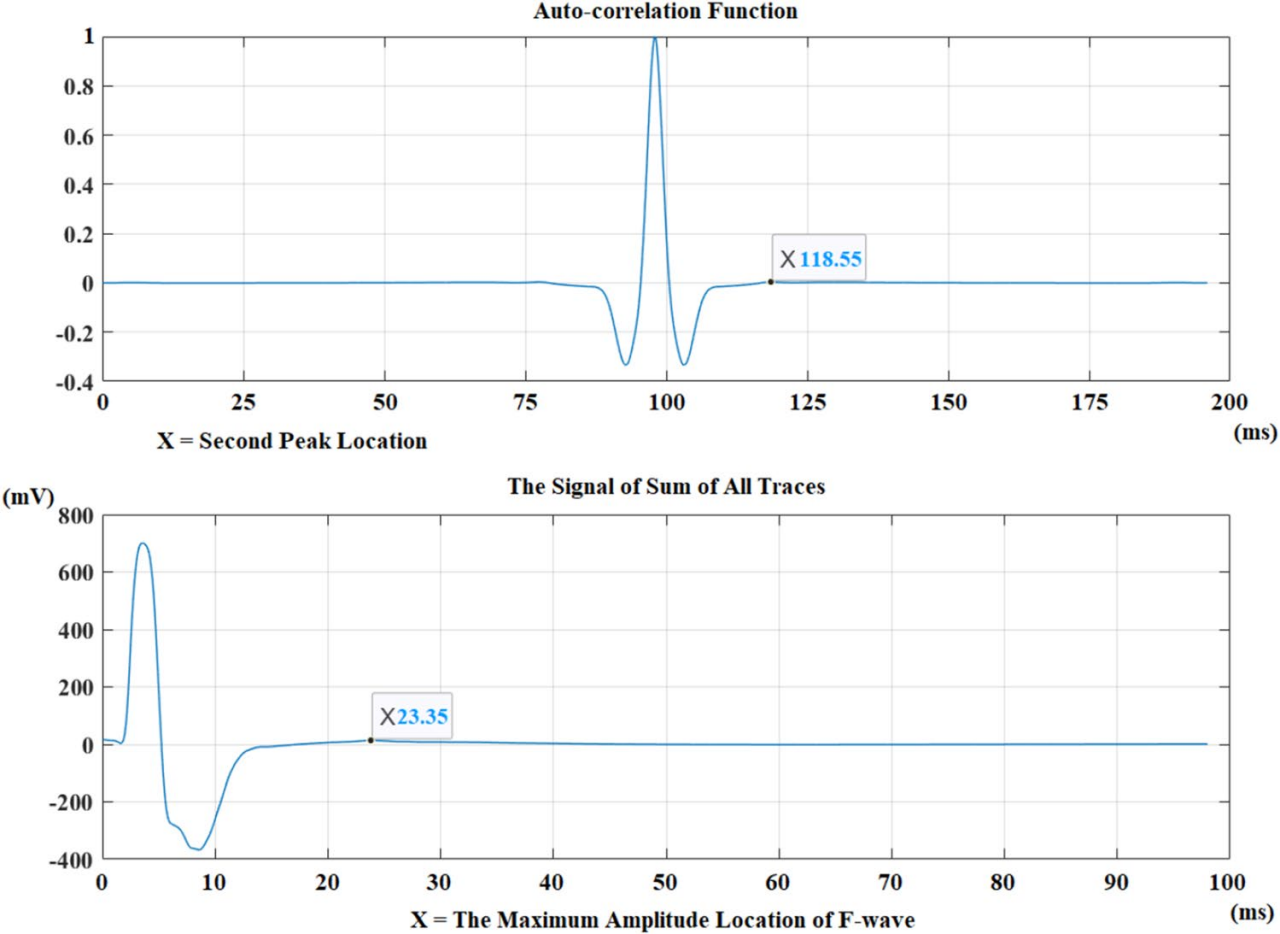
The selected peak location (X = 118.55 ms) from the autocorrelation function and the maximum amplitude location of the F-wave (X = 23.35 ms) for a healthy control from a median nerve recording.

The maximum amplitude location of the F-waves on the signal of sum of all traces and the selected peak on the autocorrelation function are shown to the user. Figure 7 shows these locations.

The maximum amplitude location for F-waves belonging to a healthy control recording from median nerve were determined as 25.35 ms. The calculated location was written as 23.35 ms at the bottom part of Fig. 7, but 2 ms should be added to find the exact maximum amplitude location of F-waves because 2 ms part has been discarded before because of the stimulus artifact.

By using a window, a signal is extracted by moving 10 ms back and by moving 20 ms forward from the maximum amplitude location of the F-waves at the signal of sum of all traces.

**Figure 8:**
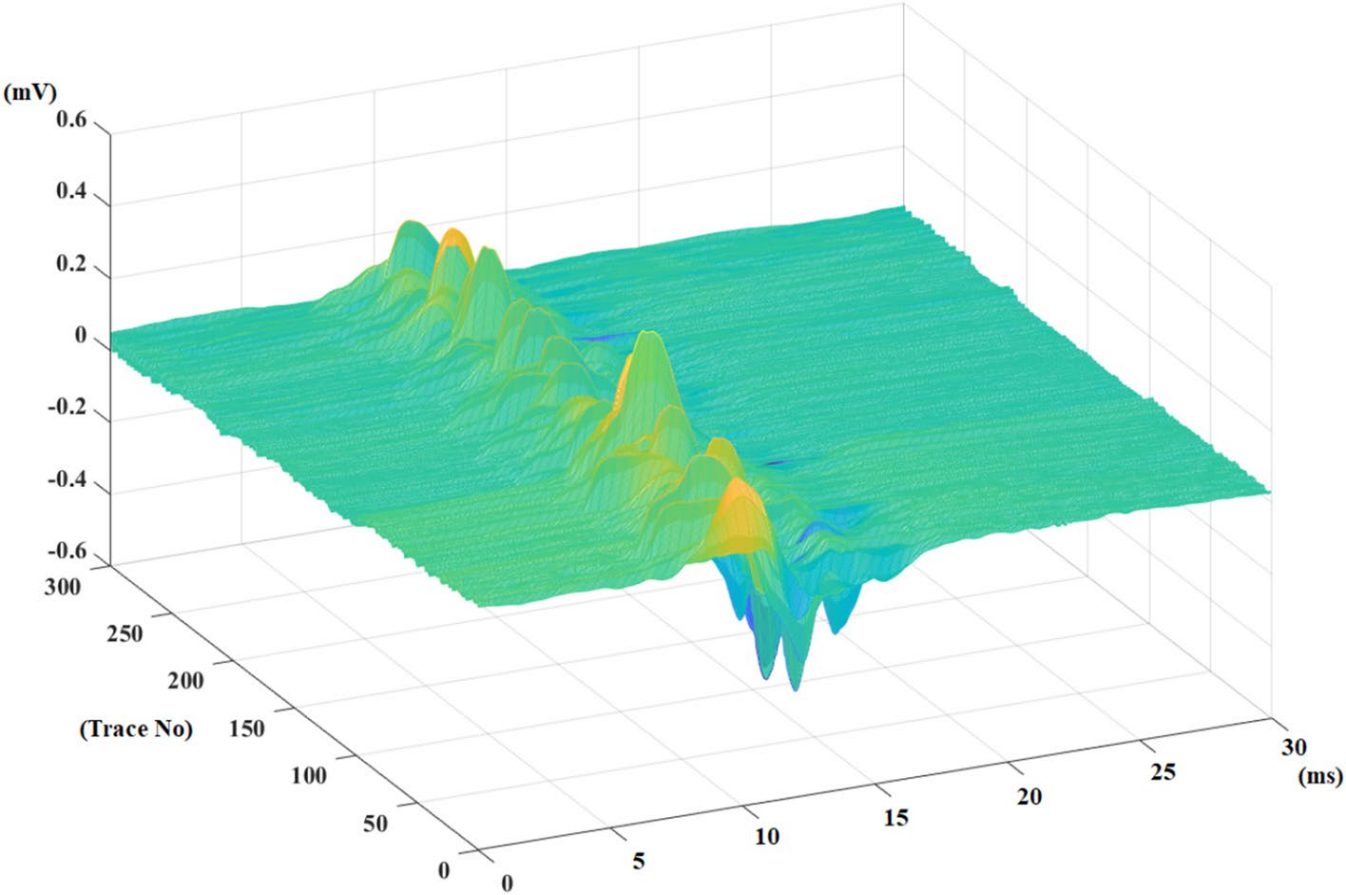
The extracted F-wave corridor from median nerve recording of a healthy control.

A 0.25 ms piece from the left of this signal is checked if its slope is greater than 16°. If the slope is greater than 16°, the left part of this signal comes from the M-response and is trimmed by the algorithm. This process is repeated until the slope of 0.25 ms pieces from the left part of the signal is lower than 16°. Then, the duration of the F-waves is printed on the screen, and all traces are cut from the calculated cutting locations. The F-wave corridor is shown in Fig. 8.

Here, the steps of manual analysis and those of automated analysis combine. The algorithm continues to flow for the next step.

After revealing the F-waves from the recording, a unique linear line is generated according to the behavior of each trace for the baseline correction process. This linear line is the estimated baseline of the original trace. It is calculated by using least squares method. When the distances of samples on the top and bottom of the linear line are summed individually, the absolute values of these two magnitudes are same.

Every sample of this unique line is subtracted from the corresponding trace point by point. At the end of this process, the angles of all traces are corrected, and they are all placed on the ‘0 reference line’. After the baseline correction process, all baselines are standardized and they all become same.

**Figure 9:**
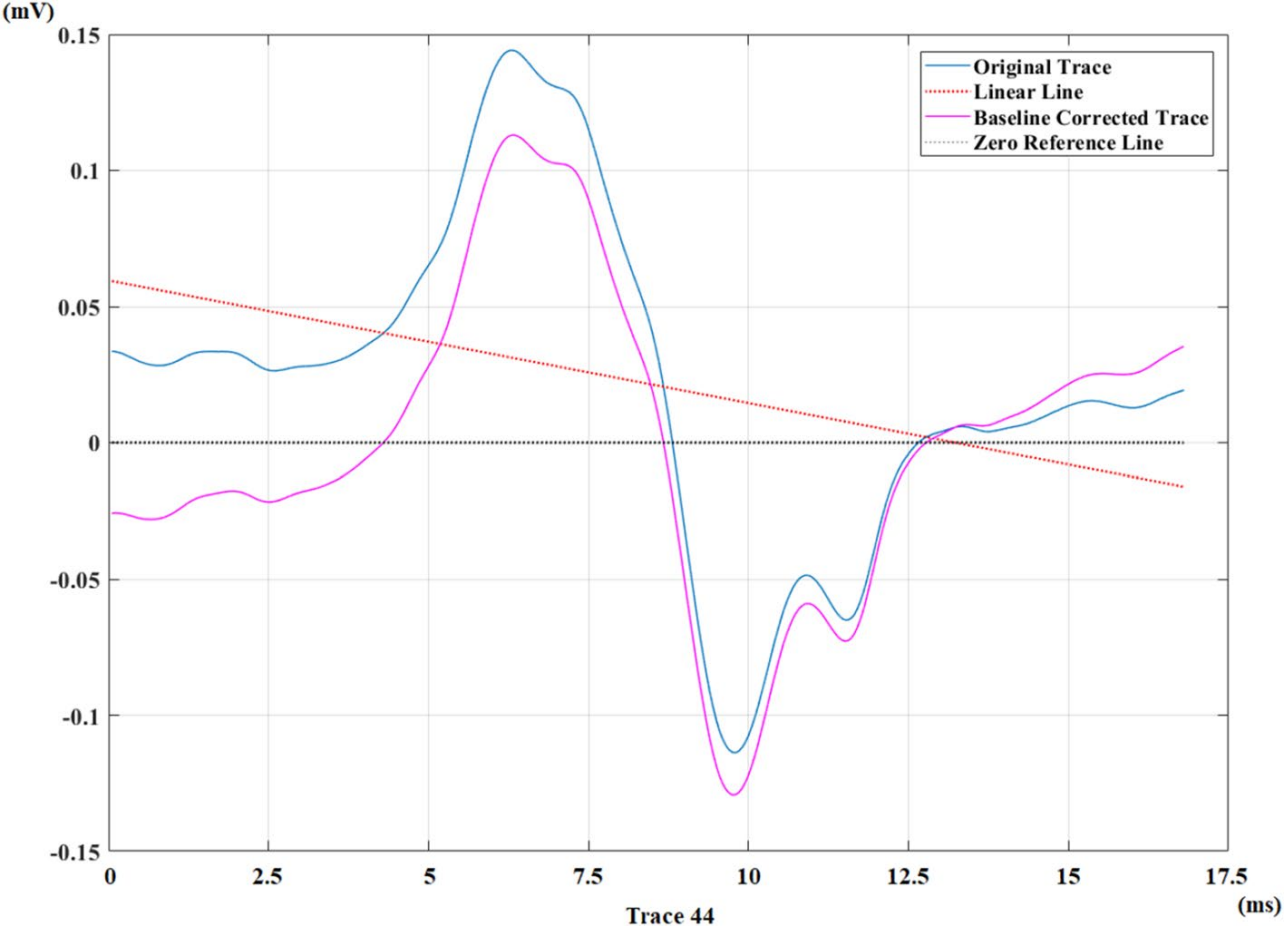
Linear line (estimated baseline) belonging to the original trace (blue colored) of a healthy control recording for the baseline correction and final result (purple colored) for that trace after the baseline correction.

Figure 9 shows the generated linear line belonging to a trace from a healthy control recording and the result after baseline correction.

**Figure 10:**
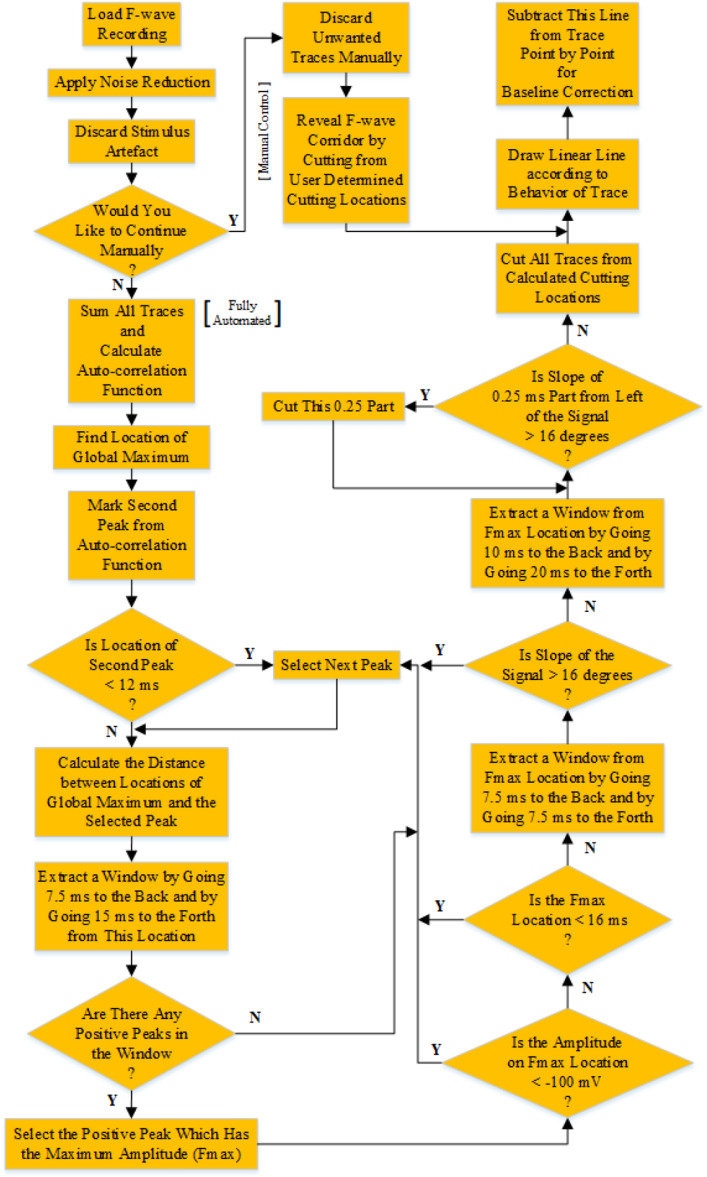
Flow chart of both the automated F-wave extraction algorithm and baseline correction part.

The flow chart of the developed algorithm up to this phase is given in Fig. 10.

The automated algorithm continues with extracting F-wave parameters. The peak-to-peak amplitude of the M-response is calculated for use in the MUNE analysis. The maximum and minimum amplitude values of the F-waves are calculated and stored separately. The peak-to-peak amplitudes of the F-waves are calculated afterward. The maximum and minimum amplitude locations of F-waves are determined for a later step in the algorithm.

As stated in the electrophysiological evaluation part, the amplitudes of F-waves are checked to determine if they satisfy the 40 µV criterion. If the amplitude of any F-wave is below 40 µV, it is marked and discarded from analysis.

The fall time of a trace is lower than 3 ms. If the amplitude change of a trace is lower than 40 µV in 3 ms from the peak location to the left and right, it is evaluated as a noise signal. It is also marked and discarded from the analysis.

All remaining traces are aligned according to their maximum amplitude locations. For becoming a repeater F-wave pair candidate, all traces are compared with each other in terms of maximum and minimum amplitude (Fmax and Fmin) locations. Both Fmax and Fmin location differences for each repeater F-wave candidate pair should be below 0.5 ms. To maintain their candidacy, the amplitude ratio of candidates should be greater than 0.9; as a result, they satisfy amplitude similarity.

The power values of repeater F-wave candidates are calculated next. If the power ratio of the candidates is greater than 0.8, they remain as candidates. The correlation coefficient is another parameter that should be checked to satisfy the morphological similarity of repeater F-waves. If this coefficient is greater than 0.9 for candidate pairs, they maintain their candidacy.

One more control parameter was derived for approving candidates as repeater F-waves. It was named as ‘the similarity coefficient’ in a previous study^26^. The similarity coefficient consists of the sum of the amplitude difference and power difference values between the elements of a candidate pair. If this coefficient for a candidate pair is lower than 0.6, they are confirmed as repeater F-waves. This value has been determined by practicing with a great number of recordings of various types.

After these operations, the remaining F-waves are checked again in the second pass. They are aligned according to their minimum amplitude locations. All traces are compared with each other in terms of minimum amplitude locations. The Fmin location difference for each repeater F-wave candidate pair should be below 0.5 ms.

The difficulty level for candidacy criteria is increased. The amplitude ratio of candidates should be greater than 0.95, and the power ratio of the candidates should be greater than 0.9 at this time.

The similarity coefficient is calculated again with the sum of the amplitude difference and power difference values between the elements of each candidate pair. If this coefficient for a candidate pair is lower than 0.6, they are confirmed as repeater F-waves.

If one element of a repeater F-wave pair is the same as another repeater F-wave pair element, they are combined in the same group.

The number of repeater F-waves is counted, and it is the first calculated feature. The number of repeater F-wave groups is named as ‘the number of repeater neuron’ feature. The sMUP amplitude feature is calculated for each repeater F-wave group. Persistence is the ratio of the number of F-waves in the analysis to the total number of recorded traces.

Three different parameters can be used for F-MUNE calculations. These are peak-to-peak amplitude, positive peak area and total area parameters. The equations for calculating the MUNE are given as follows:

1 $${\text{MUNE}}_{1} = \frac{{{\text{M}}_{{{\text{pp}}}} \;{\text{amplitude}}}}{{\left\{ {\sum\nolimits_{{{\text{n}} = 1}}^{{\text{K}}} {({\text{sMUP}}_{{{\text{pp}}}} \;{\text{amplitude}})_{{\text{n}}} } } \right\}/{\text{K}}}}$$

2 $${\text{MUNE}}_{2} = \frac{{{\text{M positive peak area}}}}{{\left\{ {\sum\nolimits_{{{\text{n}} = 1}}^{{\text{K}}} {({\text{sMUP positive peak area}})_{{\text{n}}} } } \right\}/{\text{K}}}}$$

3 $${\text{MUNE}}_{3} = \frac{{{\text{M total area}}}}{{\left\{ {\sum\nolimits_{{{\text{n}} = 1}}^{{\text{K}}} {({\text{sMUP total area}})_{{\text{n}}} } } \right\}/{\text{K}}}}$$

where, M_pp_ = M-response peak-to-peak, sMUP_pp_ = Surface motor unit potential peak-to-peak.

**Figure 11:**
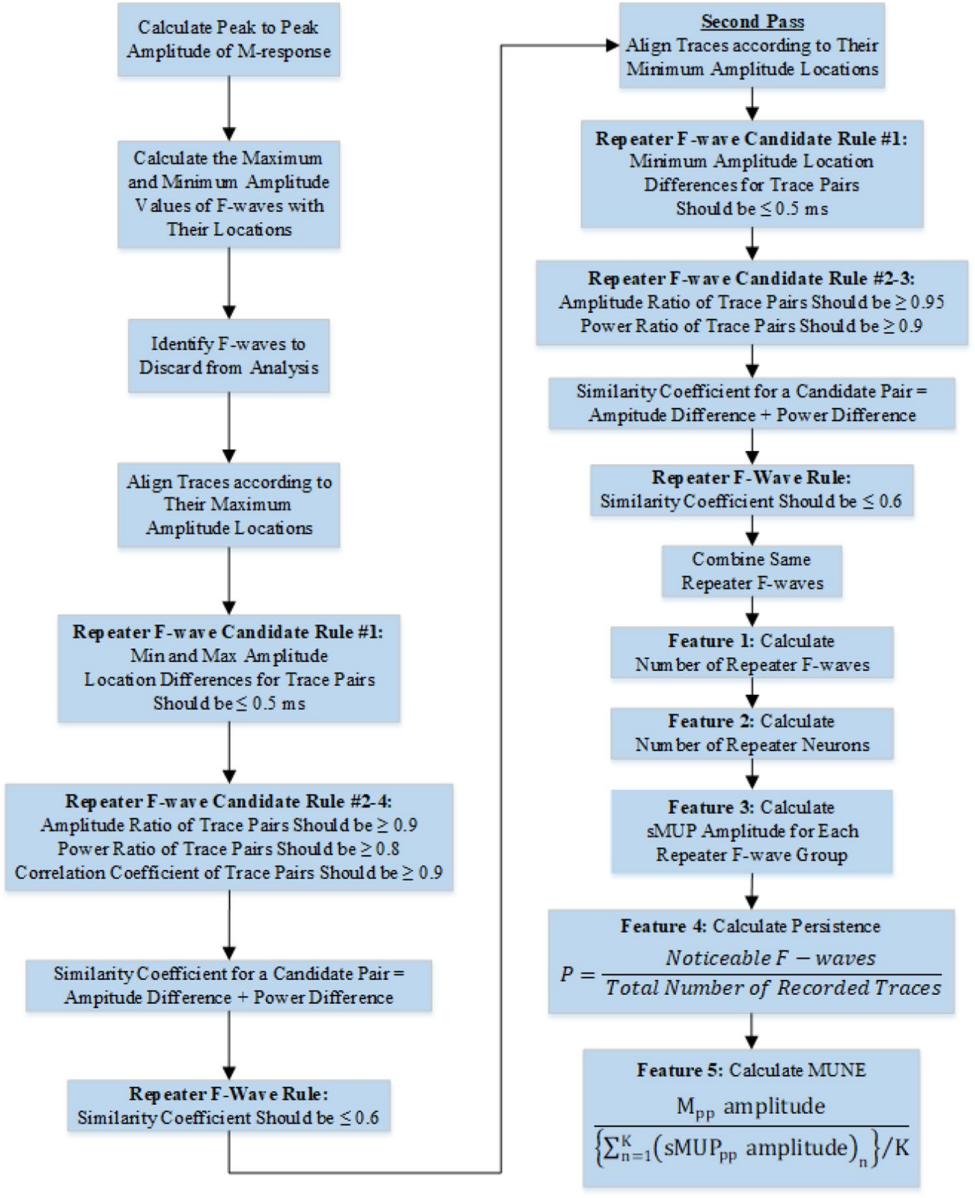
Flow chart of the feature extraction algorithm from F-waves.

The automated algorithm can perform MUNE calculations according to Eq. (1), so it is calculated by dividing peak-to-peak amplitude of M-response to mean of peak-to-peak amplitudes of sMUPs. On the other hand, it is possible to add calculation options for the other equations as an update. The flow chart of the automated algorithm for the feature extraction process from F-waves is given in Fig. 11.

**Figure 12:**
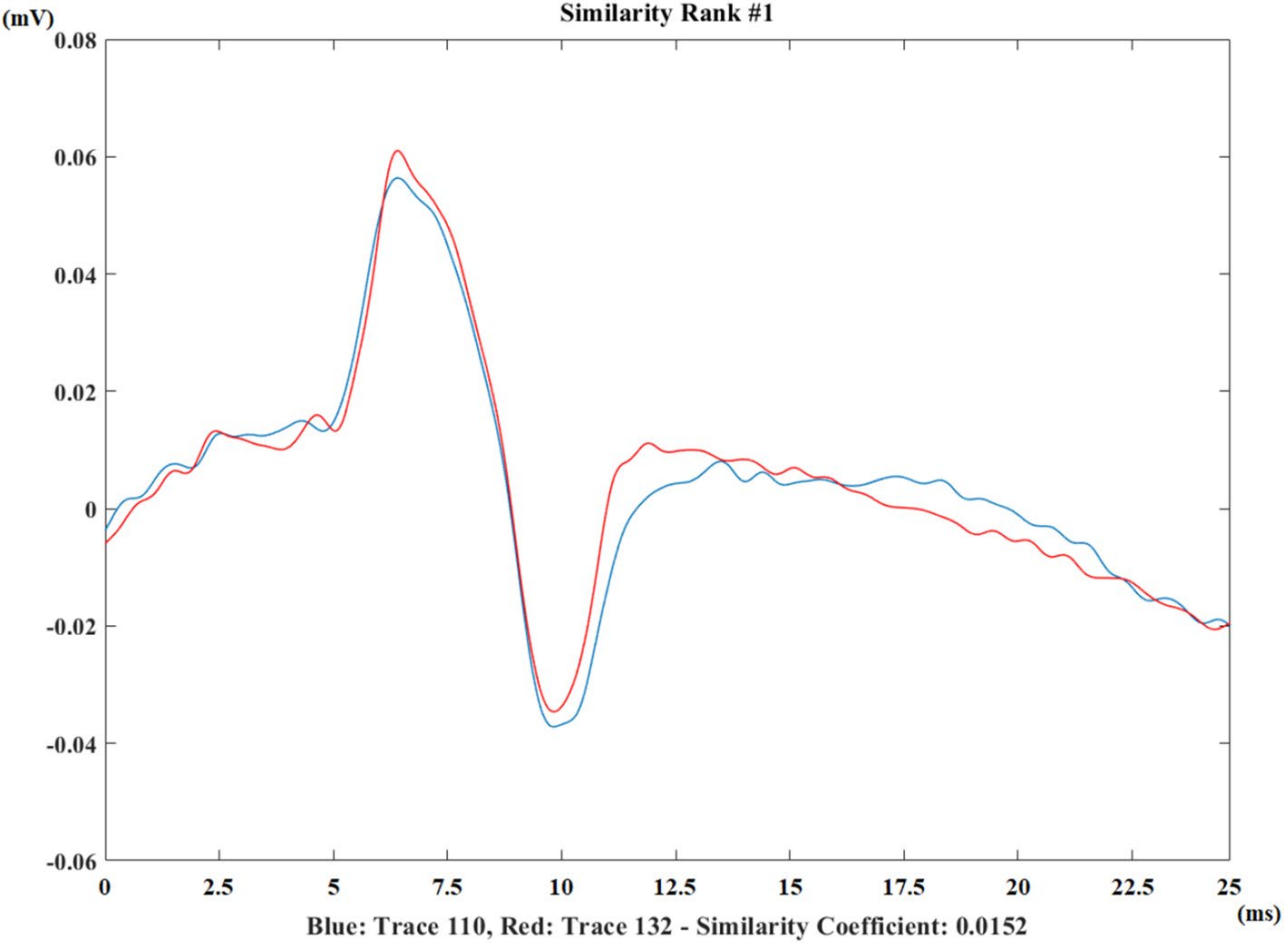
The most similar repeater F-wave pair belonging to a healthy control recording [lower similarity coefficient means more similar F-wave pairs].

A detailed report and calculation results regarding these features are presented to the user. It is possible to plot repeater F-wave pairs in overlapped way with different colors for easy distinction. In addition, a repeater F-wave pair can also be plotted under the other repeater F-wave pair. The most similar repeater F-wave pair is shown to the user. The plot of the most similar pair for a healthy control from median nerve recording is shown in Fig. 12.

## Results

All 10 recordings for each group showed repeater F-waves. After determining and discarding noise signals from the recordings, four features were calculated for the median and ulnar nerve recordings. The calculated values for these features are provided in Table 1.

**Table 1:** Calculated F-wave features.

	# Repeater neuron [mean ± STD] (min–max)	# Repeater F-wave [mean ± STD] (min–max)	Mean sMUP Amp (µV) [mean ± STD] (min–max)	MUNE [mean ± STD] (min–max)
ALS				
APB	9.3 ± 6.22 (3–19)	88.9 ± 72.61 (15–254)	345.7 ± 213.94 (122.3–912.6)	24.01 ± 10.65 (5.4–40.3)
ADM	15 ± 6.22 (9–27)	125.5 ± 74.18 (26–231)	240.94 ± 84.32 (129.8–380.2)	35.72 ± 15.39 (16.3–63.5)
Healthy controls				
APB	22.2 ± 16.08 (4–45)	90.9 ± 62.42 (8–181)	145.75 ± 32.5 (101–204.3)	84.75 ± 11.03 (69–105.4)
ADM	12.9 ± 11.67 (2–33)	58 ± 46.38 (5–137)	151.7 ± 23.54 (121.4–194.4)	65.18 ± 12.8 (49.4–84)
Polio				
APB	20.3 ± 10.76 (10–37)	107.7 ± 54.73 (35–196)	322.21 ± 108.3 (151.5–461.8)	34.66 ± 14.05 (15.4–56.1)
ADM	26 ± 12.46 (8–49)	116.4 ± 67.04 (28–239)	228.46 ± 55.81 (140.1–315.2)	40.71 ± 9.63 (27.7–51)

The mean value for the number of repeater neurons was 9.3 for ALS patients, 22.2 for healthy controls and 20.3 for polio patients in the median nerve. The same feature was calculated in the ulnar nerve as 15, 12.9 and 26 for ALS patients, healthy controls and polio patients, respectively. Logarithmic transform was applied on this feature for recordings from both nerves to make the feature normally distributed. ALS and polio patients were significantly different from each other (p < 0.05) in the median nerve. Regarding the same feature, healthy controls and polio patients were found to be significantly different from each other (p < 0.05) in the ulnar nerve.

The number of repeater F-waves was calculated to be 88.9 as the mean value in the median nerve for ALS patients. The same feature was calculated as 90.9 and 107.7 for healthy controls and polio patients, respectively. In the ulnar nerve, the mean value for this feature was 125.5 for ALS patients, 58 for healthy controls and 116.4 for polio patients. There was no significant difference between groups for this feature in both nerves.

**Figure 13:**
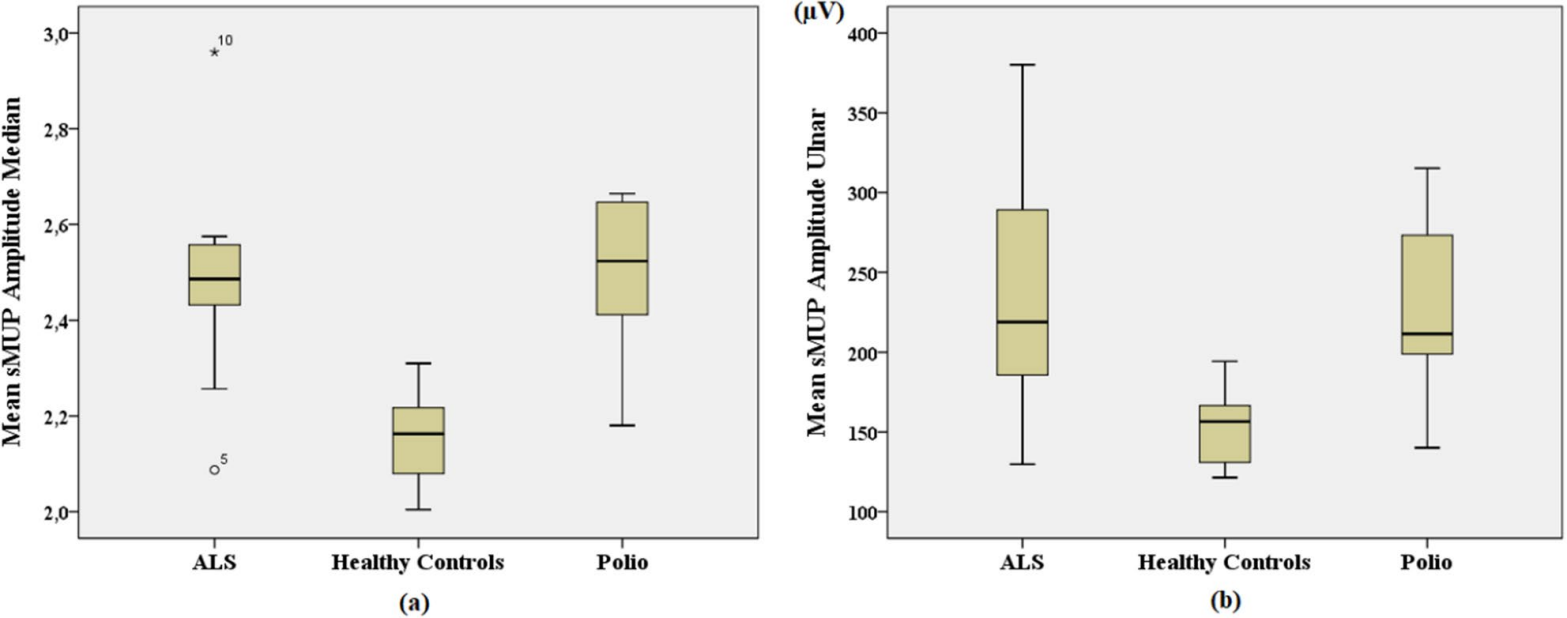
Mean sMUP amplitude values for ALS patients, healthy controls and polio patients in (**a**) median nerve (logarithmic transformed) and in (**b**) ulnar nerve (µV). *o* outlier, *extreme outlier.

In median nerve, logarithmic transform was applied to the mean sMUP amplitude feature to make it normally distributed. The mean number for this feature was calculated as 345.7 µV, 145.75 µV and 322.21 µV for ALS patients, healthy controls and polio patients, respectively. The values of the healthy controls were significantly lower than those of the patients (p < 0.05). In the ulnar nerve, the mean value for the mean sMUP amplitude feature was 240.94 µV for ALS patients. For healthy controls, this value was calculated to be 151.7 µV. Finally, the same value was calculated to be 228.46 µV for polio patients. When the values of healthy controls were compared with those of the patients, they were significantly lower (p < 0.05). Box plots for this feature are depicted in Fig. 13 for both nerves.

**Figure 14:**
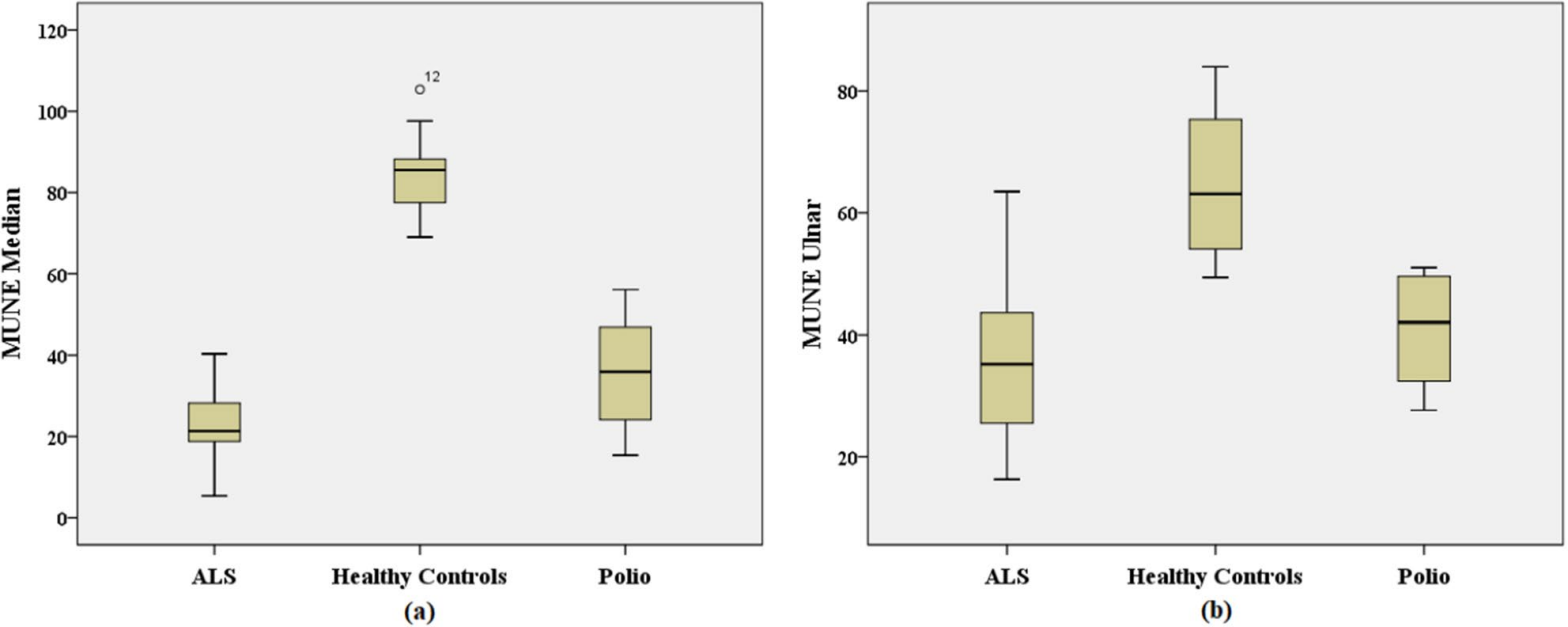
MUNE values for ALS patients, healthy controls and polio patients in (**a**) median nerve and in (**b**) ulnar nerve. *o* outlier.

The mean MUNE value in the median nerve for ALS patients was 24.01. This value was 84.75 and 34.66 for healthy controls and polio patients, respectively. In the ulnar nerve, the mean value for MUNE was 35.72 for ALS patients, 65.18 for healthy controls and 40.71 for polio patients. Healthy controls were found to be significantly different from patients according to the results for both the median and ulnar nerves (p < 0.05). Figure 14 shows the box plot for the MUNE value belonging to all groups for both recording sites.

The performance of the algorithm for automatically determining the cutting locations for F-waves was examined. An expert neurophysiologist determined the mean left and right cutting locations for polio patients at median nerve recordings as 20.87 ms and 51.28 ms. For the same group, the ulnar nerve recordings mean cutting locations were given as 20.33 ms and 49.6 ms. The algorithm calculated these values for the median nerve recordings as 20.98 ms and 49.8 ms. The related mean values for the ulnar nerve recordings were 19.95 ms and 48.65 ms.

The cutting locations for ALS patients were specified at the median nerve recordings as 21.38 ms and 51.92 ms by the expert neurophysiologist. The same values for the ulnar nerve recordings were 22.41 ms and 48.11 ms, respectively. The algorithm calculated cutting locations for ALS patients at median nerve recordings were 21.64 ms and 51.59 ms. For the ulnar nerve recordings, the locations were calculated to be 20.59 ms and 50.42 ms.

The same expert neurophysiologist determined that the mean left and right cutting locations for healthy controls at the median nerve recordings were 22.2 ms and 42.98 ms. The mean cutting locations at the ulnar nerve recordings were specified to be 22.34 ms and 43.56 ms. The algorithm calculated that the mean cutting locations at the median nerve recordings were 18.70 ms and 48.50 ms. The locations of interest for the ulnar nerve recordings were calculated to be 18.47 ms and 47.75 ms, respectively, by the developed algorithm.

## Discussion

In the current study, mathematical methods were explained for the proper extraction of F-waves from traces. The pre-processing steps were important to achieve better results. Redundant data were discarded from the text file, which contained data from the recordings, and noise reduction was applied to all traces.

The autocorrelation function was the key to finding the cutting locations of the F-wave corridor. Positive peaks from the autocorrelation function gave approximate locations for the maximum amplitude location of F-waves. This location can be found by using this information at the signal of sum of all traces.

In addition to the possibility of manual control, the algorithm was fully automated and could decide if the calculated location was correct or not. When the location was wrong, it could be updated automatically.

After revealing F-waves by using the calculated cutting locations, all F-waves were subjected to baseline corrections. Linear line estimation was preferred for correcting the baseline of all traces. The purpose of this preference was to prevent waveform distortion. The newly developed algorithm was tested with the recordings belonging to healthy controls, ALS patients and polio patients.

The number of repeater neurons, number of repeater F-waves, mean sMUP amplitude and MUNE value were calculated for all groups. The mean sMUP amplitude and MUNE value were important F-wave parameters for differentiating healthy controls from patients. The significance of these two parameters can be seen in Figs. 13 and 14.

Chroni et al.^16^ studied healthy controls, as in the current study. The number of volunteers was similar like in the current study. They recorded 40 traces in each session, while there were 300 traces in the current study. The recording muscle was the same, and they calculated the number of repeater neurons as 17 while they were using submaximal stimuli (60%). In the current study, the same value was calculated to be 12.9. This mild difference might be due to the number of recorded traces and the criteria for accepting signals. They used their own developed algorithm to perform these calculations. Their algorithm allowed manual control over the software, while the developed algorithm in the current study was fully automated with the possibility of manual control.

Veltista et al.^19^ continued their study^16^ and introduced their ‘F-wave Analyzer’ software. Their algorithm could calculate all F-wave parameters and allow manual control for changing the markers of F-waves. The currently developed algorithm was intentionally fully automated to offer simplicity to neurophysiologists, but it is also possible to perform manual control on the algorithm.

Their amplitude criteria for accepting an F-wave for analysis was lower (20 µV) than that (40 µV) in the current study. While evaluating F-waves, in their study, they used the term ‘similarity index’ which is similar to the term ‘similarity coefficient’ in our previously published study^28^.

They made their recordings from ADM muscle and included ALS patients and healthy controls in their subject pool as in the current study. Submaximal stimuli were preferred in this study, unlike using supramaximal stimuli, as in their study. The number of volunteers in their study was higher (52) than the current study (10). Forty traces were recorded in their study, while 300 traces were preferred in the current study.

The mean number of repeater neurons was 15 for ALS patients and 12.9 for healthy controls in the current study, while their calculated values for the same parameter were 4.44 and 1.27, respectively.

In the current study, the calculated value for the mean number of repeater F-waves was 125.5 for ALS patients and 58 for healthy controls. However, their calculated values were 15.19 for ALS patients and 2.73 for healthy controls. Even though the differences between these results seem large, when the percentages of the mean number of repeater F-waves to all traces were taken into account, they appear similar, especially for ALS patients (38% and 41.8% for their study and for the current study, respectively).

The mean amplitude values for repeater F-waves were 240.94 µV and 151.7 µV in the current study for ALS patients and healthy controls, respectively. In their study, the same values were calculated to be 404 µV and 384 µV. This difference might be the result of the F-wave acceptance criteria, stimulation method and number of recorded traces. They did not calculate the MUNE value, so it was not possible to perform a comparison. They also reported that 5 min are required for analysis with their developed software.

The algorithm of the current study enables a rapid analysis leading to a better performance that can be valuable, especially in cases where high numbers of traces were acquired. The present software can perform all F-wave parameter calculations in less than 2 (usually < 1) minutes. A summary of the detailed comparison between previous studies and the current study is presented in Table 2.

**Table 2:** Comparison of the parameters for the current study with those of previous studies.

References	# Traces	Subjects	Muscle	Amplitude criteria (µV)	Stimulation type	# Repeater neurons	MUNE	Calculation time
Chroni et al.^16^	40	Healthy controls	ADM	> 25	Submaximal (60%)	17	–	–
Veltista et al.^19^	40	Healthy controls	ADM	> 20	Supramaximal	1.27	–	~ 5 min
Veltista et al.^19^	40	ALS	ADM	> 20	Supramaximal	4.4	–	~ 5 min
Current study	300	Healthy controls	ADM	> 40	Submaximal (30–50%)	12.9	65.18	< 2 min (usually < 1)
Current study	300	Polio	ADM	> 40	Submaximal (30–50%)	26	40.71	< 2 min (usually < 1)
Current study	300	ALS	ADM	> 40	Submaximal (30–50%)	15	35.72	< 2 min (usually < 1)

The newly developed algorithm offers fully automated analysis with the possibility of manual control for F-wave parameters. It provides neurophysiologists the opportunity to focus on the results by decreasing the burden of manual analysis. It enriches the documentation with additional graphics.

Linear baseline estimation improves the performance of the algorithm in terms of the analysis. If the baseline of the traces is not in a standard form, the algorithm might make an incorrect decision. Repeater F-waves can only be detected correctly with the help of baseline corrections.

Qian et al.^21^ used wavelet transform feature points to draw a baseline with interpolation. Davidson et al.^29^ used a moving average to correct curvilinear baselines. In contrast to the methods in other studies, linear baseline estimation is better for F-wave studies because potentials harbor valuable information. It is important to reserve the original signal while performing manipulations without causing any distortion.

In addition, MUNE analysis can also be performed as an add-on function with this algorithm. Lastly, studies on polio patients in terms of F-wave parameters to date are scarce. However, the current study becomes prominent by the fact that polio patients were also evaluated.

On the other hand, in muscles located near the spinal cord, because the F-wave elicits so close to the global minimum of the M-response, the algorithm may not work properly. Therefore, it is not possible to analyze every muscle because of the nature of F-waves^30^.

## Conclusion

A fully automated algorithm with the possibility of manual control was developed to perform rapid and objective F-wave studies. This algorithm could find the maximum amplitude location of F-waves by using an autocorrelation function and the signal of sum of all traces. Then, F-waves were revealed by extraction from traces to perform F-wave studies. Baseline corrections were applied on all traces after the cutting process. Linear line estimation was preferred to avoid causing any distortion in the signals.

Well-known F-wave parameters such as the number of repeater neurons, number of repeater F-waves, persistence and mean sMUP amplitude can be calculated. Additional graphics provided the examiner with documentation. Furthermore, the MUNE value could be calculated as well. All calculations could be performed in nearly 1 min while evaluating 300 traces.

F-wave parameters belonging to polio patients were compared with healthy controls and ALS patients. Previously, there were a few number of F-wave studies on polio patients. This study contributed to the literature by sharing the results of the features belonging to polio patients. The mean sMUP amplitude and MUNE value were valuable features for differentiating healthy controls from patients.

The developed algorithm offers simplicity to the examiner by not requiring manual control, so only the analysis results can be focused on. More recordings from different muscles of both the upper and lower extremities will be evaluated to test the accuracy and performance of the software. In the next version of the algorithm, new graphics will be added to enrich the presentation.

## Data Availability

The data acquired during the current study are not publicly available due to privacy/ethical restrictions but are available from the corresponding author on reasonable request.
